# Relationship between Polycystic Ovarian Syndrome and Subsequent Gestational Diabetes Mellitus: A Nationwide Population-Based Study

**DOI:** 10.1371/journal.pone.0140544

**Published:** 2015-10-21

**Authors:** Mei-Lien Pan, Li-Ru Chen, Hsiao-Mei Tsao, Kuo-Hu Chen

**Affiliations:** 1 Institute of Information Science, Academia Sinica, Taipei, Taiwan; 2 Department of Physical Medicine and Rehabilitation, Mackay Memorial Hospital, Taipei, Taiwan; 3 Department of Mechanical Engineering, National Chiao-Tung University, Hsinchu, Taiwan; 4 Institute of Biomedical Informatics, National Yang-Ming University, Taipei, Taiwan; 5 Department of Obstetrics and Gynecology, Taipei Tzu-Chi Hospital, The Buddhist Tzu-Chi Medical Foundation, Taipei, Taiwan; 6 School of Medicine, Tzu-Chi University, Hualien, Taiwan; John Hopkins University School of Medicine, UNITED STATES

## Abstract

**Objective:**

This nationwide population-based study aims to explore the relationship between polycystic ovarian syndrome (PCOS) and subsequent gestational diabetes mellitus (GDM).

**Methods:**

Data from 1998–2012 Taiwan National Health Insurance Research Database were used for this study. ICD9-CM codes 256.4X and 648.X were used separately for the diagnoses of PCOS and GDM, which were further confirmed by records of blood tests or ultrasonography to ensure the accuracy of the diagnoses. Women diagnosed at < 15 or > 45 years of age, and those diagnosed with overt diabetes mellitus or GDM prior to PCOS were excluded. During pregnancy, each woman with a previous diagnosis of PCOS was age-matched to 10 women without PCOS. Odds ratios (ORs) for risk of GDM were calculated by logistic regression analysis with adjustment for economic status and co-morbidities.

**Results:**

Among 7,629 eligible women with a valid PCOS diagnosis, 3,109 (42.87%) had subsequent pregnancies. GDM occurred frequently among women with a history of PCOS as compared to those without PCOS (20.46% vs. 10.54%, *p*<0.0001). Logistic regression analysis revealed that PCOS was associated with GDM (adjusted OR = 2.15; 95% CI:1.96–2.37). Among 3,109 affected patients, 1,160 (37.31%) had used medications for PCOS and 261 (8.39%) were treated with an oral hypoglycemic agent (OHA). There was no significant difference in development of GDM between the medication and no medication sub-groups (*p*>0.05). If not used after conception, OHAs did not reduce the risk of GDM (adjusted OR = 1.20; 95% CI:0.88–1.62).

**Conclusions:**

A history of PCOS is a significant and independent risk factor for development of GDM. Medication for PCOS or pre-pregnancy use of OHAs does not reduce the risk of GDM. When at-risk women become pregnant, they require closer surveillance for maternal and fetal well-being, and should follow a strict diet and adhere to weight gain control to avoid obstetric complications due to GDM.

## Introduction

Polycystic ovary syndrome (PCOS), the most common endocrine disorder affecting women during the reproductive years, is a syndrome of ovarian dysfunction characterized by hyperandrogenism, chronic anovulation, and typical morphologic changes of the ovaries based on ultrasonographic examination [1–4]. The prevalence of PCOS is estimated to be approximately 5%-14% of reproductive-aged women [2,3], and affected patients often present with symptoms and signs of menstrual irregularity, obesity, infertility, and androgen excess [4]. Approximately 50% of women with PCOS have co-existing metabolic syndrome [5,6], in whom insulin resistance is a common endocrine disorder and the risk of developing type 2 overt diabetes mellitus (DM) is 5-8-fold over women without PCOS [6,7].

In contrast to overt DM, gestational diabetes mellitus (GDM) is defined as impaired glucose tolerance that is induced by pregnancy, perhaps from exaggerated physiologic changes in glucose metabolism [4,8]. The reported prevalence of GDM varies between 0.2% and 20% of pregnancies depending on the screening method, gestational age, and the population studied [4,9,10]. Maternal and fetal effects of uncontrolled GDM include stillbirths, macrosomia, and birth trauma [4]. Although the association between PCOS and overt DM is well-established, there is a lack of large-scale or population-based studies exploring the relationship between PCOS and GDM. Because of the similarity in the associated metabolic disturbances between PCOS and GDM, it has been postulated that there might be a connection between the two pathologies [1,4]. Some studies have reported that PCOS is associated with insulin resistance and GDM [11–19], and remains an independent predictor for GDM (risk odds ratio: 1.9–2.94) [12,13,16], while other studies have shown that the prevalence of GDM is not elevated in women with a history of PCOS [20–22]. Indeed, the conflicting conclusions drawn from these studies with different samples and different research designs are confusing. Nevertheless, the major limitation in common in previous studies is that most were conducted using small samples with < 50 PCOS patients and a method of purposive sampling, which makes the conclusions questionable and less powerful. Furthermore, many of the studies were conducted using physician-identified inclusion criteria for PCOS. Subjective identification of the PCOS diagnosis made by physicians, rather than a stricter standard of PCOS diagnosis (i.e., the Rotterdam criteria [23]), may result in selection bias. Moreover, most studies did not exclude patients with pre-existing overt DM before a diagnosis of GDM was made. This limitation will have a large confounding effect on the results of these studies. It is therefore important to clarify the role of PCOS as a predictor of future GDM using a large nationwide population-based sample with stricter selection criteria to reach a reliable conclusion.

## Materials and Methods

### Data Source and Sample

The National Health Insurance (NHI) program in Taiwan was launched in 1995. The NHI covered 93% of the population in Taiwan in 1997, and increased to 99% coverage by the end of 2010. The National Health Insurance Research Database (NHIRD) is a nationwide database extracted from the claim data of the NHI program for research purposes. This anonymous database contains information regarding inpatient and outpatient medical claims, including prescription records. In this database, ICD9-CM codes are used for disease diagnosis and NHI codes are used for treatments and procedures. In this study, we used a longitudinal cohort dataset (Longitudinal Health Insurance Database [LHID2010]) that contains claim data of one million randomly sampled individuals who were insured in 2010. This dataset has been confirmed to have no significant differences in either age, gender, or health care costs from the entire population composed of all beneficiaries under the NHI program.

### Inclusion and Exclusion Criteria

We selected women who were first diagnosed with PCOS (ICD9 code: 256.4X) between 1998 and 2012 from LHID2010. If the diagnosis did not accompany blood tests for LH, FSH, or testosterone (NHI codes: 09078B2, 09126B, 09126C, 09078B1, 09125B, 09125C, 09064B2, 09121B, and 09121C) or ultrasonography (NHI code: 19003C), the diagnosis was not considered valid. Because this study aimed to analyze the relationship between PCOS and GDM, we focused on women who were of reproductive age during the study period. Thus, women with PCOS diagnosed at < 15 or > 45 years of age were excluded. Furthermore, women who were diagnosed with overt DM (ICD9 code: 250.X) or GDM (ICD9 code: 648.X) prior to PCOS were also excluded.

In the database of NHIRD, we selected women during 1998–2012, when the diagnosis of GDM was made based on tranditional 3-hour 100 gm oral glucose tolerance test (OGTT) rather than new 75 gm OGTT. This means that all pregnant women who failed to pass 50 gm glucose tolerance test were arranged 100 gm OGTT. Abnormal blood values for a 3-hour 100 gram OGTT are defined as fasting: greater than 105 mg/dL, 1 hour: greater than 190 mg/dL, 2 hour: greater than 165 mg/dL, and 3 hour: greater than 145 mg/dL. If more than 1 of above blood glucose results was higher than normal, GDM was diagnosed. Although there is a lack of worldwide consensus about the diagnosis of PCOS until 2003 when Rotterdam criteria for PCOS were established, in Taiwan the diagnosis of PCOS was consistently made if physicians noted more than one of the following changes including ovarian dysfunction, hyperandrogenism and polycystic ovary (PCO) morphology. Based on findings of the symptoms, ultrasonography and labortory tests, the diagnosis and coding for PCOS are stricter and can be more believable. During the study period, the criteria for the diagnosis of PCOS and GDM remained unchanged in Taiwan.

Because GDM is a condition that occurs during pregnancy, we had to identify women with PCOS who had subsequent pregnancies. In the current study, women who have ever been pregnant were defined as those who had medical records documenting a vaginal delivery (ICD9 code: 650 or NHI codes: 81017C, 81024C, 81034C, 97001A, 97001K, 97002A, 97003B, 97004C, 97005D, 97931K, 97932A, 97933B, and 97934C), cesarean delivery (ICD9 codes: 652.X, 654.21, 653.X, and 669.71 or NHI codes: 81004C, 81005B, 81005C, 81028C, 81029C, 97006K, 97007A, 97008B, 97009C, 97014C, 98001K, 98002A, 98003B, and 98004C), multifetal pregnancy (ICD9 code: 651.X or NHI codes: 81018C, 81019C, 81025C, and 81026C), preterm labor (ICD9 codes: 644.03 and 644.21), prenatal visit (ICD9 code: V22.X), or induction after 24 weeks gestation (NHI code: 81010C). Women with PCOS and subsequent pregnancies were designated as the case group.

**Fig 1:**
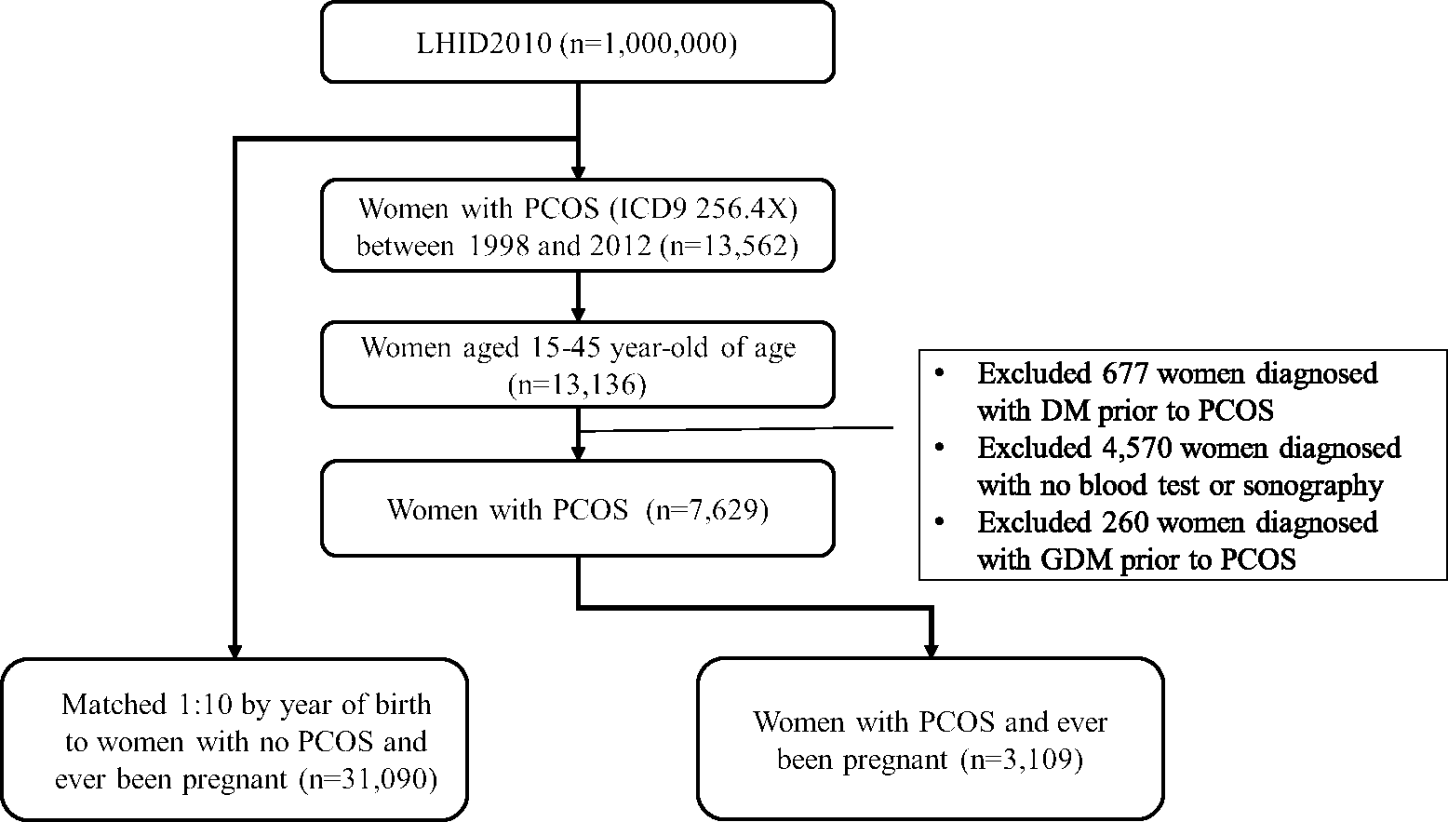
The flow chart of case selection and exclusion of women with a history of PCOS.

In the current study GDM was confirmed by a diagnosis of ICD9 code 648.X during prenatal visits, which are covered under the NHI program in Taiwan. During regular prenatal visits, a routine OGTT is scheduled at 24–28 weeks gestation. Hence, using NHIRD for the diagnosis and calculation of GDM is feasible and verifiable. Because it is rare that pregnant women do not receive prenatal visits in Taiwan, we excluded women who had no records of prenatal visits within 5 months before a diagnosis of GDM was made. As mentioned above, women who had ever been diagnosed with overt DM or whose age at the time of diagnosis of PCOS was < 15 or > 45 years were also excluded to ensure the accuracy of diagnosis. We then created a control group from the remaining women in the LHID2010. During pregnancy, each woman with a history of PCOS (the case group) was matched to 10 women without a history of PCOS (the control group) by age (year of birth). The flow chart of case selection and exclusion is shown in Fig 1.

In addition to assessing the relationship between PCOS and subsequent GDM, we further analyzed whether or not medical treatment for PCOS has any effect on preventing the future development of GDM. The medications for PCOS are mainly categorized into three types, including oral hypoglycemic agents (OHAs), ovulation inducers (clomiphene), and cyproterone. In the treatment of PCOS, cyproterone is the first-line drug that is commonly used for reduction of androgen level to improve symptoms induced by hyperandrogenism, rather than for the goal of contraception. Although use of cyproterone may therefore impact phenotyping or general appearance, such effect on general appearance may be not associated with future GDM and use of cyproterone should not be excluded in the study. However, other oral contraceptives that is used for the goal of contraception rather than for the treatment of PCOS may be a confounder in this study, and should be excluded. In the current study, OHA refers to metformin, α-glucosidase inhibitors, dipeptidyl peptidase 4 (DPP-4) inhibitors, sulfonamides, and thiazolidinediones. We then categorized patients with the diagnosis of PCOS into two sub-groups according to medication use. The patients who had ever used OHAs, ovulation inducers, or cyproterone were defined as the medication sub-group. Finally, we categorized women with the diagnosis of PCOS into OHA use and no OHA use sub-groups to analyze the sole effect of OHA use.

### Ethics Statement

All claim data in the research database were anonymized and de-identified before release. According to the local regulations, informed consent was not required for the current study. This study was approved by the Institutional Review Board of Taipei Tzu-Chi Hospital, Taiwan (approval number: 02-XD21-064).

### Data analysis

SAS^®^ (version 9.3; SAS Institute, Inc., Cary, NC, USA) was used for all data analyses. We analyzed the general characteristics, such as age at the time of initial diagnosis of PCOS, economic status, and co-morbidities, among the case and control groups. Odds ratios (ORs) for the risk of GDM were calculated by logistic regression analysis with adjustment for confounding factors, including economic status and co-morbidities. In the current study we used insurable wage as a proxy measurement of economic status. The classification of economic status was represented by insurable wage in the NHI system (current exchange rate is US$ 0.0321 = NTD 1.00). Thus, we divided economic status into 4 groups, as follows: insurable wage < NTD 20,000; NTD 20,000–40,000; ≧ NTD 40,000; and other (spouse/dependents). ICD9-CM codes for co-morbidities, one of the possible confounding factors, were listed as follows: hypertension (401.X-405.X); dyslipidemia (272.X); congestive heart failure (428.X); coronary artery disease (414.X); cerebrovascular disease (430.X-438.X); and chronic pulmonary disease (490.X-496.X). A *p*-value < 0.05 was set as the level of significance.

## Results

According to the selection and exclusion criteria, we identified 7,629 eligible women with valid PCOS diagnoses in this study (Fig 1). Among these women, 3,109 (42.87%) had subsequent pregnancies. Comparisons of the characteristics between the case and control groups are listed in Table 1. The “age” in Table 1 represented the age of the patients when PCOS was initially diagnosed (three groups of age 15–25, 26–35, and 36–45). During the study, every patient with PCOS (case group) was matched to another woman of the same age (control group). For the case group, the mean age at the time of initial diagnosis of PCOS was 29.06 ± 5.4 years. Most patients (62.5%) were initially diagnosed with PCOS between 26 and 35 years of age. Compared with the control group, women in the case group had a higher percentage of insurable wage ≧ NTD 40,000 (19.88%), a lower percentage of insurable wage < NTD 20,000 (22.23%), and spouse/dependents (15.31%). Overall, the economic status was significantly different between the case and control groups (*p* < 0.0001). Compared with the control group, women in the case group were more likely to have dyslipidemia (*p* < 0.0001) and a higher economic status (*p* < 0.0001; Table 1).

**Table 1:** Characteristics of women with and without a history of PCOS.

	Case Group: women with PCOS (n = 3,109)		Control Group: women without PCOS (n = 3,109)		Statistics		
	N	%	N	%	OR	95% CI	*p*-value
**AGE** (mean ± SD)	29.06(5.4)		29.06(5.4)				1.0000
15–25	804	25.86	8,040	25.86			
26–35	1,943	62.50	19,430	62.50			
36–45	362	11.64	3,620	11.64			
**Economic status**							<0.0001*
Insurable wage <$20000 NTD	691	22.23	7,689	24.73			
Insurable wage $20000–40000 NTD	1,324	42.59	13,346	42.93			
Insurable wage > = $40000 NTD	618	19.88	4,740	15.25			
Other (Spouse/dependents)	476	15.31	5,315	17.1			
**Co-morbidities**							
Hypertension	68	2.19	544	1.75	1.26	0.96–1.62	0.0794
Dyslipidemia	180	5.79	1,056	3.40	1.75	1.48–2.06	<0.0001*
Congestive heart failure	6	0.19	50	0.16	1.20	0.42–2.80	0.6395
Coronary artery disease	8	0.26	43	0.14	1.86	0.76–4.01	0.1352
Cerebrovascular disease	53	1.70	435	1.40	1.22	0.90–1.63	0.1770

Data are expressed as the number (%) or mean ± standard deviation, as appropriate

*p < 0.0001, by chi-square test or student t test, as appropriate

The risk analysis of the relationship between PCOS and subsequent GDM is listed in Table 2. We demonstrated that GDM occurred frequently among women with a history of PCOS as compared to women without PCOS (20.46% vs. 10.54%, *p* < 0.0001). After adjustment, logistic regression analysis revealed that PCOS is a significant and independent risk factor for GDM (adjusted OR = 2.15; 95% CI: 1.96–2.37). Among 3,109 patients with PCOS, 1,160 patients (37.31%) used medications for PCOS. There was no significant difference in the development of subsequent GDM between the medication and no medication sub-groups (*p* > 0.05). We further determined whether or not OHA use for the treatment of PCOS reduced the risk of developing GDM in comparison to no OHA use. Among 3,109 patients with PCOS, only 264 (8.49%) patients received OHA treatment. Overall, OHAs did not reduce the risk of GDM (adjusted OR = 1.20; 95% CI: 0.88–1.62) if not used after conception.

**Table 2:** Risk analysis of the relationship between PCOS and subsequent GDM.

	No GDM		GDM		Statistics	
	N	%	N	%	Crude OR (95% CI)	Adjust OR (95% CI)*
**Group**						
Control Group: women without PCOS (n = 31,090)	27,814	89.46	3,276	10.54	Reference	Reference
Case Group: women with PCOS (n = 3,109)	2,473	79.54	636	20.46	2.18 (1.98–2.40)	2.15 (1.96–2.37)
**Sub-group in Case Group**						
No Medication Group (n = 1,949)	1,566	80.35	383	19.65	Reference	Reference
Medication Group (n = 1,160)	907	78.19	253	21.81	1.14 (0.95–1.36)	1.14 (0.96–1.37)
**Sub-group in Case Group**						
No OHA use (n = 2,845)	2,271	79.82	574	20.18	Reference	Reference
OHA use (n = 264)	202	76.52	62	23.48	1.19 (0.88–1.61)	1.21 (0.90–1.64)

Odds ratio (OR) and 95% confidence intervals (95% CI) are calculated by logistic regression analysis, as compared to the reference group

*Adjusted for economic status and dyslipidemia

## Discussion

The results of this study revealed that women with a previous diagnosis of PCOS are associated with a higher incidence of GDM in future pregnancies as compared to women without PCOS (20.46% vs. 10.54%, p<0.0001). Further analysis showed that pre-existing PCOS is a significant and independent risk factor (adjusted OR = 2.15; 95% CI: 1.96–2.37) for GDM. Women with a history of PCOS have a higher risk of GDM in future pregnancies, and require closer surveillance for maternal and fetal well-being. Appropriate information and suggestions should be provided to at-risk pregnant women after confirmation of pre-existing PCOS to facilitate earlier intervention or referral. When affected women become pregnant, they may consider following a strict diet and adhering to weight gain control to avoid obstetric complications due to GDM.

There is one possible and reasonable explanation for our findings. In addition to classical symptoms and signs, including irregular menstruation, obesity, infertility, and a higher serum level of androgen, PCOS is associated with increased insulin resistance, which may remain unimproved and may be responsible for the occurrence of GDM in future pregnancies; however, the result of this study showed that OHA treatment (metformin) alone or a combination of PCOS drugs (clomiphene or cyproterone acetate) plus OHA did not reduce the occurrence of GDM for women with a previous history of PCOS. The overall findings are somewhat inconsistent with previous reports, which indicated that using metformin in pregnant women with a history of PCOS might reduce the occurrence of GDM [24] or the requirement of insulin use for existing GDM [25].

Our study showed that all women that were already taking metformin or other OHAs for PCOS (at any time after the diagnosis was made) did not have a lower incidence of GDM than women with PCOS who were not taking PCOS-related medication. This result actually implied that not many of the women with PCOS were really taking OHAs after pregnancy (given that metformin amongst others is a treatment for GDM). Some patients with PCOS might discontinue the use of OHAs after their conceptions. We have re-analyzed the result by further dividing PCOS patients with OHAs use into those who used before and those who used after conception. A total of 264 patients with PCOS had recieved OHA medication. Of them, 146 patients used OHAs before conception (and discontinued OHA use after conception) and 118 patients used OHAs after conception. Among the former (146 patients), 46 patients (31.51%) was diagnosed as having GDM; among the latter (118 patients), 16 patients (13.56%) was diagnosed as having GDM. There was a significant difference of outcome between using OHA before and after conception (p = 0.001). Compared to the incidence (20.18%) of GDM in PCOS patients who did not use OHAs, there were a higher incidence (31.51%) of GDM for patients who used OHAs before conception and a lower incidence (13.56%) of GDM for patients who used OHAs after conception. This indicated that the severity of PCOS and the goal of OHAs use may be different between PCOS patients who used OHA before and after conception. A possible interpretation for this analysis of sub-grouping is that the patients who needed OHA medication before conception might have a higher severity of PCOS, thus had a higher incidence of GDM in the future. On the other hand, patients who used OHA medication after conception had a lower incidence (13.56%) of GDM. The may be attributed to that the use of OHA medication could lower the level of blood sugar before or when an OGTT was performed in the 2nd trimester. A detailed discussion of this possible mechanism is beyond the scope of our study, but we believe that further efforts can be made to explore the etiology that underlies GDM in women with pre-existing PCOS as well as relevant medication. Overall, even if our study showed that all women that were already taking metformin or other OHAs for PCOS (at any time) did not have a lower incidence of GDM than women with PCOS who were not taking PCOS-related medication, the use of OHA (metformin) should not be stopped after pregnancy if necessary.

The study herein has a number of strengths, including a relatively large sample size, sampling method, and stricter selection criteria of patients for the diagnosis of PCOS or GDM. To the best of our knowledge this is the first study using a nationwide population for assessment of the relationship between PCOS and GDM. An advantage of the study is that the sample is retrieved from a database of a general survey of a nationwide population rather than from purposive sampling. The sample size of the study is the largest among all studies of its kind to investigate the prevalence of GDM for women with an existing diagnosis of PCOS. Compared with a meta-analysis that summarizes 15 qualified studies (649 PCOS patients and 4,466 controls) [12], our study has a larger sample size (3,109 PCOS patients and 31,090 controls). Our results are therefore robust due to minimization of possible errors that originate from the sampling process. Additionally, all diagnoses for patients with PCOS or GDM were made by objective examinations of blood tests or pelvic ultrasonography [23] rather than by a physician’s subjective judgment. All bias resulting from the samples, the investigator, the selection, and the measuring process were minimized as much as possible by the methods and the criteria used.

This study is the only nationwide study that examines the association between PCOS and GDM; however, it has some inherent limitations that need to be concerned. First, it is not easy to precisely define a medical condition from administrative data. Using ICD9 codes alone it is unable to reflect the real status of clinical conditions. Herein, we not only used ICD9 codes to identify cases, but also included hormone testing, blood testing, and ultrasonography as essential inclusion criteria to raise the appropriateness of the case definition. Second, administrative data were collected for the purpose of reimbursement under the NHI. Therefore, the existing data may be inconsistently collected over time. This may have affected the findings to some extent. Third, some demographic characteristics are lacking in NHIRD, such as body mass index (BMI), marital status, and social status. Thus, we were unable to investigate the contributions of these factors. It’s not possible to obtain the data because body weight and height of every resident were not routinely recorded in this nationwide health database of Taiwan. Moreover, although the power of the analysis of sub-grouping is enough (more than 100 patients for each sub-group), we could not afford to obtain the information of dosage and duration regarding the use of OHA medication, which was a congenital and inevitable limitation of the national database, and might affect the result of the study. Finally, due to the ethnic homogeneity in Taiwan, this study cannot tackle the ethnic effect on the association between PCOS and GDM.

## Conclusions

From this nationwide population-based study, we have demonstrated that pregnant women with a history of PCOS have a more than two-fold increased probability of GDM compared with women without PCOS. Medication for PCOS or pre-pregnancy use of OHAs does not reduce the risk of GDM. We recommend that obstetricians should be more aware of the increased risk of subsequent GDM in women with a history of PCOS. When at-risk women become pregnant, they should follow a stricter diet and adhere to weight gain control to avoid obstetric complications due to gestational diabetes.

## References

[1] Kousta E, Cela E, Lawrence N, Penny A, Millauer B, White D, et al The prevalence of polycystic ovaries in women with a history of gestational diabetes. Clin Endocrinol. 2000;53: 501–507. 10.1046/j.1365-2265.2000.01123.x 11012576

[2] Hart R. Polycystic ovarian syndrome—prognosis and treatment outcomes. Curr Opin Obstet Gynecol. 2007;19: 529–535. 18007129 10.1097/GCO.0b013e3282f10e22

[3] Dunaif A. Insulin resistance and the polycystic ovary syndrome: mechanism and implications for pathogenesis. Endocr Rev. 1997;18: 774–800. 9408743 10.1210/edrv.18.6.0318

[4] Eroglu D, Zeyneloglu HB. Metabolic disorders in patients with recent gestational diabetes mellitus. J Obstet Gynaecol Res. 2006;32: 408–415. 16882267 10.1111/j.1447-0756.2006.00418.x

[5] Ehrmann DA, Barnes RB, Rosenfield RL, Cavaghan MK, Imperial J. Prevalence of impaired glucose tolerance and diabetes in women with polycystic ovary syndrome. Diabetes care. 1999;22: 141–146. 10333916 10.2337/diacare.22.1.141

[6] Glintborg D, Henriksen JE, Andersen M, Hagen C, Hangaard J, Rasmussen PE, et al Prevalence of endocrine diseases and abnormal glucose tolerance tests in 340 Caucasian premenopausal women with hirsutism as the referral diagnosis. Fertil Steril. 2004;82: 1570–1579. 15589862 10.1016/j.fertnstert.2004.06.040

[7] Legro RS, Dunaif A. The Role of Insulin Resistance in Polycystic Ovary Syndrome. Endocrinologist. 1996;6: 307–321.

[8] Cunningham FG, Leveno KJ, Bloom SL, Hauth JC, Gilstrap LC III, Wenstrom KD. Diabetes In: Cunningham FG, editors. Williams Obstetrics. New York: McGraw-Hill; 2005 pp. 1172–1173.

[9] Miehle K, Stepan H, Fasshauer M. Leptin, adiponectin and other adipokines in gestational diabetes mellitus and pre-eclampsia. Clin Endocrinol. 2012;76: 2–11. 10.1111/j.1365-2265.2011.04234.x 21951069

[10] Holte J, Gennarelli G, Wide L, Lithell H, Berne C. High prevalence of polycystic ovaries and associated clinical, endocrine, and metabolic features in women with previous gestational diabetes mellitus. J Clin Endocrinol Metab. 1998;83: 1143–1150. 9543131 10.1210/jcem.83.4.4707

[11] Kashanian M, Fazy Z, Pirak A. Evaluation of the relationship between gestational diabetes and a history of polycystic ovarian syndrome. Diabetes Res Clin Pract. 2008;80: 289–292. doi: 10.1016/j.diabres.2007.12.022 18294722 10.1016/j.diabres.2007.12.022

[12] Boomsma CM, Eijkemans MJ, Hughes EG, Visser GH, Fauser BC, Macklon NS. A meta-analysis of pregnancy outcomes in women with polycystic ovary syndrome. Hum Reprod Update. 2006;12: 673–683. 16891296 10.1093/humupd/dml036

[13] Lo JC, Feigenbaum SL, Escobar GJ, Yang J, Crites YM, Ferrara A. Increased prevalence of gestational diabetes mellitus among women with diagnosed polycystic ovary syndrome: a population-based study. Diabetes care. 2006;29: 1915–1917. 16873802 10.2337/dc06-0877

[14] Weerakiet S, Srisombut C, Rojanasakul A, Panburana P, Thakkinstian A, Herabutya Y. Prevalence of gestational diabetes mellitus and pregnancy outcomes in Asian women with polycystic ovary syndrome. Gynecol Endocrinol. 2004;19: 134–140. 15697074 10.1080/09513590400007242

[15] Bjercke S, Dale PO, Tanbo T, Storeng R, Ertzeid G, Abyholm T. Impact of insulin resistance on pregnancy complications and outcome in women with polycystic ovary syndrome. Gynecol Obstet Invest. 2002;54: 94–98. 12566751 10.1159/000067719

[16] Mikola M, Hiilesmaa V, Halttunen M, Suhonen L, Tiitinen A. Obstetric outcome in women with polycystic ovarian syndrome. Hum Reprod. 2001;16: 226–229. 11157811 10.1093/humrep/16.2.226

[17] Radon PA, McMahon MJ, Meyer WR. Impaired glucose tolerance in pregnant women with polycystic ovary syndrome. Obstet Gynecol. 1999;94: 194–197. 10432126 10.1016/s0029-7844(99)00252-5

[18] Anttila L, Karjala K, Penttila RA, Ruutiainen K, Ekblad U. Polycystic ovaries in women with gestational diabetes. Obstet Gynecol. 1998;92: 13–16. 9649084 10.1016/s0029-7844(98)00133-1

[19] Paradisi G, Fulghesu AM, Ferrazzani S, Moretti S, Proto C, Soranna L, et al Endocrino-metabolic features in women with polycystic ovary syndrome during pregnancy. Hum Reprod. 1998;13: 542–546. 9572407 10.1093/humrep/13.3.542

[20] Haakova L, Cibula D, Rezabek K, Hill M, Fanta M, Zivny J. Pregnancy outcome in women with PCOS and in controls matched by age and weight. Hum Reprod. 2003;18: 1438–1441. 12832369 10.1093/humrep/deg289

[21] Turhan NO, Seckin NC, Aybar F, Inegol I. Assessment of glucose tolerance and pregnancy outcome of polycystic ovary patients. Int J Gynaecol Obstet. 2003;81: 163–168. 12706273 10.1016/s0020-7292(03)00003-1

[22] Vollenhoven B, Clark S, Kovacs G, Burger H, Healy D. Prevalence of gestational diabetes mellitus in polycystic ovarian syndrome (PCOS) patients pregnant after ovulation induction with gonadotrophins. Aust N Z J Obstet Gynaecol. 2000;40: 54–58. 10870780 10.1111/j.1479-828x.2000.tb03167.x

[23] Rotterdam ESHRE/ASRM-Sponsored PCOS consensus workshop group. Revised 2003 consensus on diagnostic criteria and long-term health risks related to polycystic ovary syndrome (PCOS). Hum Reprod. 2004;19: 41–47. 14688154 10.1093/humrep/deh098

[24] Begum MR, Khanam NN, Quadir E, Ferdous J, Begum MS, Khan F, et al Prevention of gestational diabetes mellitus by continuing metformin therapy throughout pregnancy in women with polycystic ovary syndrome. J Obstet Gynaecol Res. 2009;35: 282–286. 19708174 10.1111/j.1447-0756.2008.00876.x

[25] Nawaz FH, Khalid R, Naru T, Rizvi J. Does continuous use of metformin throughout pregnancy improve pregnancy outcomes in women with polycystic ovarian syndrome? J Obstet Gynaecol Res. 2008;34: 832–837. doi: 10.1111/j.1447-0756.2008.00856.x 18834342 10.1111/j.1447-0756.2008.00856.x

